# Immunogenic Tumor Cell Death Induced by Neoadjuvant Chemotherapy in Patients With Muscle-Invasive Bladder Cancer: An Immunohistochemical Analysis

**DOI:** 10.7759/cureus.102672

**Published:** 2026-01-30

**Authors:** Tatsuki Miyamoto, Makito Miyake, Nobutaka Nishimura, Yuki Oda, Takuto Shimizu, Takuya Owari, Kota Iida, Kiyohide Fujimoto

**Affiliations:** 1 Department of Urology, Nara Medical University, Kashihara, JPN

**Keywords:** calreticulin, high-mobility group box 1, immunogenic tumor cell death, muscle-invasive bladder cancer, neoadjuvant chemotherapy

## Abstract

Aim: To investigate immunogenic cell death (ICD) induced by platinum-based neoadjuvant chemotherapy (NAC) and its impact on survival outcomes in patients with muscle-invasive bladder cancer (MIBC) who underwent radical cystectomy (RC).

Methods: This study included 45 patients with MIBC who underwent RC with (n = 25) or without NAC (n = 20). Induction of ICD in MIBC was assessed by comparing immunohistochemical analyses of two ICD-related proteins, high-mobility group box 1 (HMGB1) and calreticulin, between transurethral resection (TUR) and matched RC specimens. Clinicopathologic data, including the upregulation of ICD-related proteins, were correlated with survival outcomes after RC.

Results: Upregulation of HMGB1 and calreticulin from TUR to matched RC specimens was observed in 11 (44%) and 12 (48%) of 25 NAC-treated patients, respectively, whereas it was detected in only two (8%) and one (4%) patients without NAC (P = 0.03 and P = 0.01, respectively). Upregulation of HMGB1 was significantly associated with longer cancer-specific survival (CSS) and overall survival (P < 0.01 and P = 0.02, respectively). Upregulation of HMGB1 and histological subtype (P = 0.03 and P = 0.05, respectively) were independent prognostic factors for CSS. Furthermore, among NAC-treated patients with recurrent disease, upregulation of HMGB1 tended to correlate with a better response to subsequent immune checkpoint inhibitor therapy and longer survival (P = 0.07).

Conclusions: Our findings suggest that platinum-based NAC induces ICD in MIBC. Upregulation of HMGB1 may serve as a marker of chemotherapy-induced ICD and as a potential prognostic biomarker after RC.

## Introduction

Bladder cancer (BC) is the 10th most common malignancy and the second most prevalent urologic cancer worldwide. Approximately 70% of patients are diagnosed with non-muscle-invasive bladder cancer, whereas 30% present with muscle-invasive bladder cancer (MIBC) [1,2]. Radical cystectomy (RC) is the standard treatment for localized MIBC, and neoadjuvant chemotherapy (NAC) is recommended for appropriately selected patients. Randomized clinical trials have demonstrated that patients with localized MIBC who receive NAC before RC achieve better survival outcomes than those who undergo RC alone [3,4]. After cisplatin-based NAC, pathologic complete response has been reported in 33-42% of RC specimens, which is associated with improved survival [3,5,6]. However, even among patients who achieved a clinical complete response on transurethral resection of the bladder tumor (TURBT) after NAC and subsequently underwent RC, up to 60% had residual disease, and their oncologic outcomes varied [7].

Cell death is a natural physiological process that maintains tissue homeostasis by balancing cell production and elimination. During normal physiological activity, the immune system is frequently exposed to dead cells, not only in the context of injury or infection but also under normal conditions, and must distinguish between immunogenic and tolerogenic forms of cell death [8]. Immunogenic cell death (ICD) is a regulated form of cell death that can trigger an adaptive immune response in an immunocompetent host, often through apoptotic pathways. This response can be modulated by chemotherapeutic agents, oncolytic viruses, physicochemical therapies, photodynamic therapy, or radiation therapy [9,10]. Apoptotic cells release molecules that act as adjuvants or danger signals for the immune system. These signals, collectively known as damage-associated molecular patterns (DAMPs), include the translocation of calreticulin from the cytosol to the plasma membrane and its release into the extracellular space, as well as the release of high-mobility group box 1 (HMGB1) from the nucleus into the extracellular environment [11-13]. ICD involves the release of DAMPs from dying tumor cells, which are recognized by innate pattern recognition receptors such as toll-like receptors and NOD-like receptors. This recognition activates tumor-specific immune responses, contributing to the long-term efficacy of anticancer therapies [9,10].

Although substantial progress has been made in elucidating the cellular and molecular mechanisms of various forms of cell death over the past decades, the relationship between ICD and chemotherapy in urothelial carcinoma (UC) remains unclear. Therefore, this study aimed to clarify the induction of ICD by NAC and its association with immune markers in patients who underwent RC. Furthermore, we evaluated the relationship between ICD and cancer-specific survival (CSS) and overall survival (OS). This article was previously presented at the 110th Annual Meeting of the Japanese Urological Association on April 20, 2023.

## Materials and methods

Study cohort and patient selection

This retrospective, single-center study was approved by the Ethics Committee of Nara Medical University (reference ID: 2891) and conducted in accordance with the ethical principles of the Declaration of Helsinki (2013). Written informed consent was obtained from all participants. We reviewed 215 consecutive patients with pathologically confirmed UC who underwent RC at our institution between February 2010 and March 2022.

The exclusion criteria were as follows: (i) patients whose transurethral resection (TUR) specimens were unavailable (n = 145) and (ii) patients whose pathological tissue after RC was classified as T0 or Ta (n = 25), resulting in a total of 45 patients (21%) with MIBC included in the final analysis. Clinicopathological and follow-up data were obtained by reviewing patients’ medical records. Follow-up evaluations were performed according to the institutional protocol [14,15]. CSS and OS were defined as the intervals from the date of RC to the date of death.

Immunohistochemical staining of bladder cancer tissues

Immunohistochemical staining for HMGB1 and calreticulin was performed on formalin-fixed, paraffin-embedded tissue sections (4 μm thick) obtained from TUR and RC specimens. The Histofine® SAB-PO (MULTI) Kit (Nichirei Biosciences Inc., Tokyo, Japan) was used following the manufacturer’s instructions. The tissue sections were deparaffinized in xylene and rehydrated through a graded ethanol series. Antigen retrieval was performed in an autoclave at 121°C for 20 minutes. For HMGB1 staining, an ethylenediaminetetraacetic acid buffer (pH 9.0) was used, whereas a sodium citrate buffer (pH 6.0) was used for calreticulin. Endogenous peroxidase activity was quenched by incubation in 3% hydrogen peroxide in methanol for 15 minutes at room temperature. After washing with phosphate-buffered saline (PBS), non-specific binding was blocked using the blocking reagent provided in the kit for 10 minutes. The sections were then incubated overnight at 4°C with rabbit polyclonal antibodies against HMGB1 (ab18256; Abcam, Waltham, MA, USA) and calreticulin (ab2907; Abcam), both diluted 1:5000 in antibody diluent. Following PBS washing, the sections were incubated with the secondary antibody reagent from the Histofine kit for 10 minutes and washed again with PBS. The enzyme reagent supplied with the kit was applied for five minutes and then rinsed with PBS. Immunoreactivity was visualized using a diaminobenzidine substrate solution. Finally, the sections were counterstained with Mayer’s hematoxylin, dehydrated, cleared, and mounted with coverslips.

Quantitative evaluation of HMGB1 and calreticulin protein expression

The expression of HMGB1 and calreticulin was semi-quantitatively evaluated by combining proportion and intensity scores. The histochemical score (H-score) system was used to classify specimens into four categories by multiplying the intensity score (0-3) by the percentage of stained cells (0-100%). Expression levels were categorized as follows: negative (0; H-score 0-14), weakly positive (1+; H-score 15-99), moderately positive (2+; H-score 100-199), and strongly positive (3+; H-score 200-300) [16]. For survival analysis, upregulation of HMGB1 and calreticulin was defined as an increase of ≥50 points in the H-score between TUR and matched RC specimens, as this threshold represents a biologically meaningful change in immunohistochemical expression while maintaining adequate analytical resolution and balanced group sizes for statistical analysis.

Statistical analysis

Statistical analyses were conducted using EZR, a statistical software program based on the open-source R platform (version 3.0.2), and Prism 7.00 (GraphPad Software, Inc., San Diego, CA, USA). Continuous variables are presented as medians with interquartile ranges (IQR), and categorical variables are presented as numbers and percentages. Group comparisons were performed using Fisher’s exact test, or the chi-square test, as appropriate. CSS and OS curves were generated using the Kaplan-Meier method and compared using the log-rank test. Cox proportional hazards regression models were applied for univariable and multivariable analyses to identify factors associated with CSS and OS. Statistical significance was defined as P < 0.05.

## Results

Study population and patient characteristics

Among the 45 patients included in the study, 20 (44%) and 25 (56%) were classified into the non-NAC and NAC groups, respectively. The clinicopathological characteristics of the patients are summarized in Table 1. Compared with the non-NAC group, the NAC group had significantly fewer patients with pathological N2 stage disease (3% vs. 35%, P = 0.02) and a lower median age (70 vs. 75 years, P = 0.05). Approximately half of the patients in both groups had a histological subtype. All patients who received adjuvant therapy underwent chemotherapy, and none received adjuvant immune checkpoint inhibitor therapy. The median follow-up duration from the date of RC to the last contact was 22 months (IQR, 6-90 months). During follow-up, 26 patients (58%) developed recurrent disease, 15 (33%) died of bladder cancer progression, and 24 (53%) died from all causes.

**Table 1:** Baseline clinicopathological characteristics of 45 patients according to neoadjuvant chemotherapy (NAC) status. Data are presented as median (interquartile range) or number (percent). a: t-test, b: Fisher's exact test

Variables	Total, n (%)	non-NAC group, n (%)	NAC group, n (%)	P Value
Total	45 (100)	20 (44)	25 (56)	
Age, Median (IQR)	72 (68 - 77)	75 (71 - 79)	70 (66 - 76)	0.05^a^
Sex (%)				0.14^b^
Male	36 (80)	14 (70)	22 (88)	
Female	9 (20)	6 (30)	3 (12)	
cT stage (%)				1.00^b^
2	21 (47)	9 (45)	12 (48)	
3	14 (31)	6 (30)	8 (32)	
4	10 (22)	5 (25)	5 (20)	
cN status (%)				0.10^b^
0	36 (80)	17 (85)	19 (76)	
1	4 (9)	0 (0)	4 (16)	
2	2 (4)	2 (10)	0 (0)	
3	3 (7)	1 (5)	2 (8)	
pT stage (%)				0.88^b^
is	1 (2)	1 (5)	0 (0)	
2	20 (45)	8 (40)	12 (48)	
3	14 (31)	6 (30)	8 (32)	
4	10 (22)	5 (25)	5 (20)	
pN status (%)				0.02^b^
0	31 (69)	12 (60)	19 (76)	
1	6 (13)	1 (5)	5 (11)	
2	8 (18)	7 (35)	1 (3)	
3	0 (0)	0 (0)	0 (0)	
Tissue classification (%)				0.84^b^
Urothelial carcinoma (UC)	23 (52)	10 (50)	13 (52)	
UC with squamous differentiation	9 (20)	5 (25)	4 (16)	
UC with glandular differentiation	2 (4)	1 (5)	1 (4)	
Nested subtype	2 (4)	0 (0)	2 (8)	
Other histological subtype	9 (20)	4 (20)	5 (20)	
Adjuvant therapy				0.64^b^
No	40 (89)	17 (85)	23 (92)	
Yes	5 (11)	3 (15)	2 (8)	
Cancer-specific death				0.53^b^
No	31 (69)	12 (60)	18 (72)	
Yes	15 (31)	8 (40)	7 (28)	
All-cause death				0.55^b^
No	23 (51)	9 (45)	14 (56)	
yes	22 (49)	11 (55)	11 (44)	

Expression of HMGB1 and calreticulin in bladder cancer and according to NAC regimens

**Figure 1:**
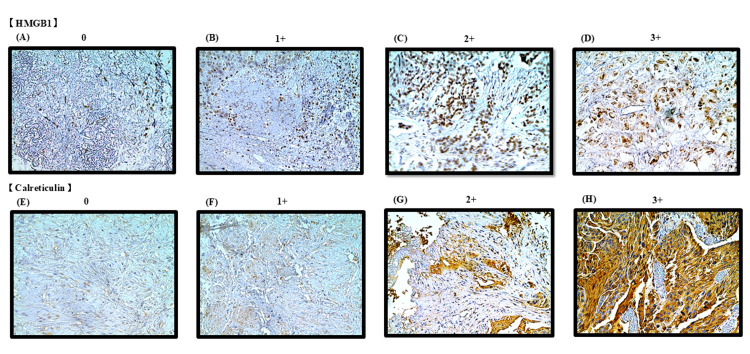
Representative histological images of high-mobility group box 1 (HMGB1) and calreticulin expression categorized by immunohistochemical scoring. All specimens were obtained from radical cystectomy samples. Representative histological images of HMGB1 and calreticulin expression categorized by immunohistochemical scoring. Pathological images (×400) of HMGB1 or calreticulin were obtained using a light microscope. (A) HMGB1-negative bladder tumor; (B) HMGB1 weakly positive bladder tumor; (C) HMGB1 moderately positive bladder tumor; (D) HMGB1 strongly positive bladder tumor; (E) calreticulin-negative bladder tumor; (F) calreticulin weakly positive bladder tumor; (G) calreticulin moderately positive bladder tumor; (H) calreticulin strongly positive bladder tumor.

**Figure 2:**
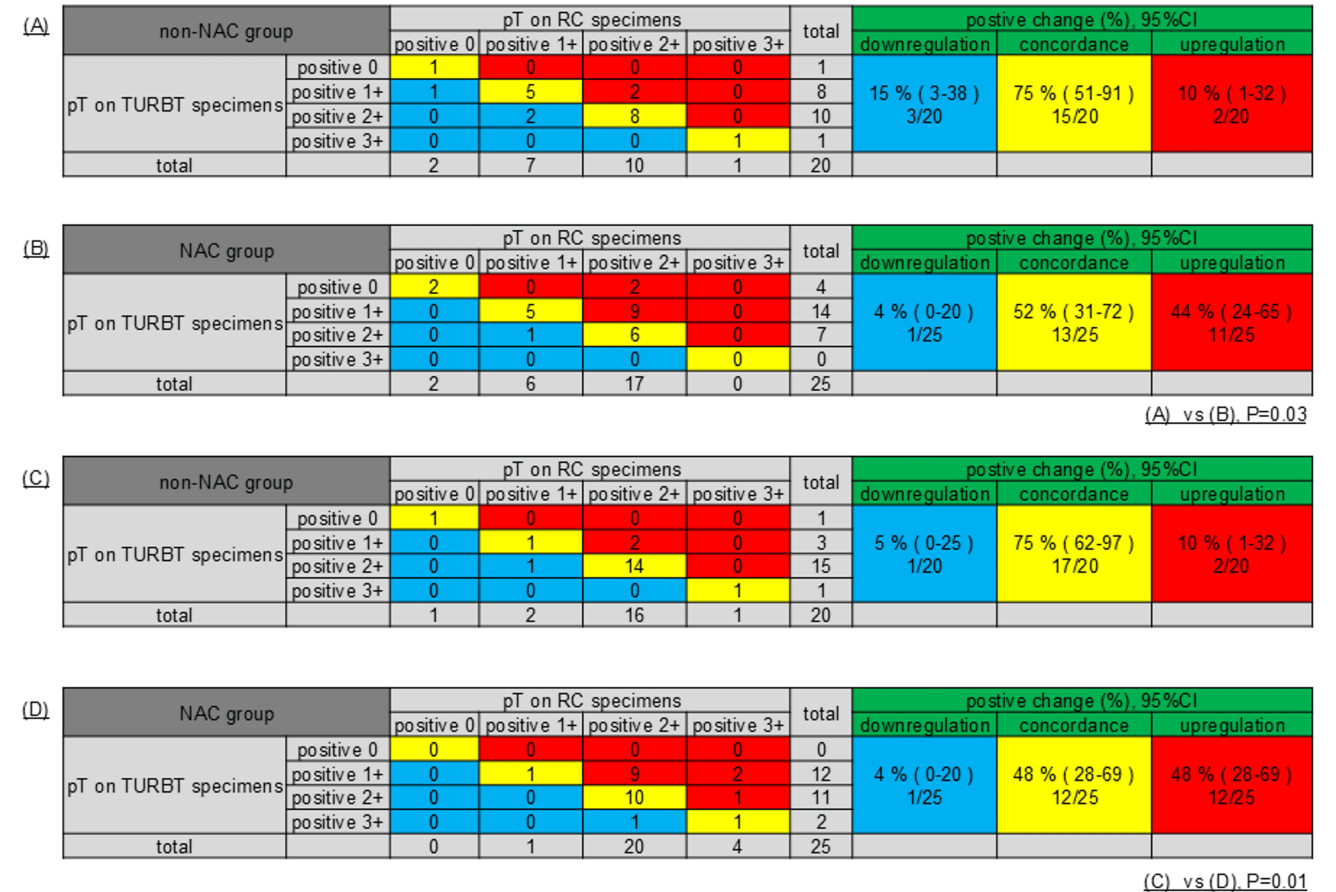
The expression of high-mobility group box 1 (HMGB1) and calreticulin was compared between transurethral resection of bladder tumor (TURBT) and radical cystectomy (RC) specimens. Tables A and B compare the expression of HMGB1, whereas Tables C and D compare the expression of calreticulin. Comparison of HMGB1 and calreticulin expression between TURBT and RC specimens. Tables (A) and (B) show HMGB1 expression, whereas tables (C) and (D) present calreticulin expression (P = 0.03 and P = 0.01, respectively). Red blocks represent the number of cases showing increased expression of HMGB1 or calreticulin when comparing TURBT specimens with RC specimens. Yellow blocks indicate cases with no change in expression, while blue blocks represent cases with decreased expression. This statistical analysis was performed using the chi-square test. NAC, neoadjuvant chemotherapy.

Representative immunohistochemical images of HMGB1 and calreticulin expression, categorized according to their H-scores, are shown in Figure 1. The expression of HMGB1 and calreticulin was compared between TUR and matched RC specimens in both the NAC and non-NAC groups. The NAC group showed significantly greater increases in HMGB1 and calreticulin expression than the non-NAC group (P = 0.03 and P = 0.01, respectively; Figure 2).

**Figure 3:**
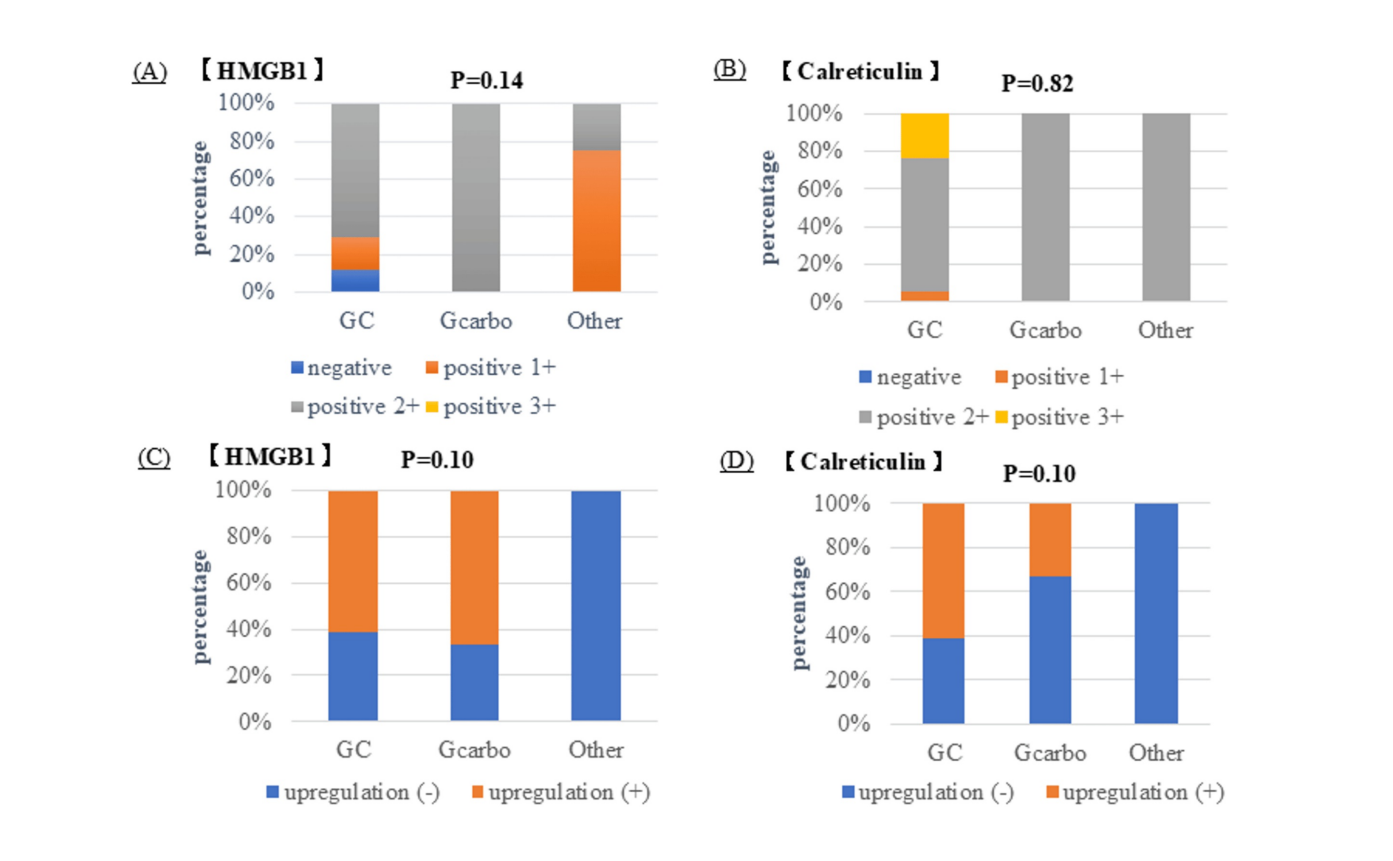
(A, B) Expression levels of high-mobility group box 1 (HMGB1) and calreticulin were evaluated for each chemotherapy regimen. (C, D) Changes in HMGB1 and calreticulin expression between transurethral resection of bladder tumor (TURBT) and radical cystectomy specimens are shown for each chemotherapy group. The other was platinum-based chemotherapy. (A, B) Expression levels of HMGB1 and calreticulin according to different neoadjuvant chemotherapy regimens. (C, D) Upregulation of HMGB1 and calreticulin between transurethral resection of the bladder tumor and radical cystectomy specimens according to chemotherapy regimen. “Other” refers to other platinum-based chemotherapy regimens. Upregulation (+) was defined as a shift to a higher expression category (e.g., from negative to weakly positive, weakly positive to moderately positive, or moderately positive to strongly positive) in the radical cystectomy specimen compared with the corresponding transurethral resection (TUR) specimen. Conversely, upregulation (−) was defined as no change or a decrease in expression category between the paired specimens. This statistical analysis was performed using the chi-square test. GC, gemcitabine plus cisplatin; GCarbo, gemcitabine plus carboplatin.

The NAC regimens consisted of gemcitabine plus cisplatin (GC) in 17 patients (68%), gemcitabine plus carboplatin (GCarbo) in four patients (16%), and other platinum-based chemotherapy regimens in four patients (16%). The expression of HMGB1 and calreticulin in TUR specimens according to the NAC regimen is shown in Figure 3A, 3B. Notably, negative HMGB1 expression was observed exclusively in patients treated with the GC regimen, whereas strong (3+) calreticulin expression was also observed only in the GC group. The expression of HMGB1 and calreticulin was compared between TUR and matched RC specimens among patients treated with NAC, and upregulation was recorded for each NAC regimen, as illustrated in Figure 3C, 3D. Upregulation was detected exclusively in the GC and GCarbo regimens, with no statistically significant difference between these regimens.

Expression of HMGB1 and calreticulin in bladder cancer

Association With ICD and Clinical Outcomes

**Figure 4:**
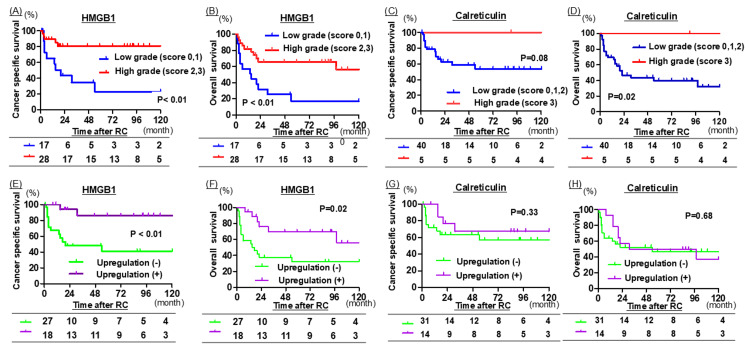
The upregulation of high-mobility group box 1 (HMGB1) and calreticulin was compared between transurethral resection of bladder tumor (TURBT) and radical cystectomy (RC) specimens. Graphs A–D: RC specimens only; Graphs E–H: both TURBT and RC specimens, all from 45 patients. (A, B) Kaplan-Meier curves for cancer-specific survival (CSS) and overall survival (OS) according to HMGB1 expression levels in RC specimens, classified into low (positivity 0–1) and high (positivity 2–3) groups. (C, D) Kaplan-Meier curves for CSS and OS according to calreticulin expression levels in RC specimens, classified into low (positivity 0–2) and high (positivity 3) groups. (E, F) Kaplan-Meier curves for CSS and OS stratified by changes in HMGB1 expression: the upregulation group (increase in HMGB1 expression from TURBT specimens to RC specimens) versus the non-upregulation group. (G, H) Kaplan-Meier curves for CSS and OS stratified by changes in calreticulin expression: the upregulation group (increase in calreticulin expression from TURBT specimens to RC specimens) versus the non-upregulation group. Upregulation (+) of HMGB1 and calreticulin was defined as an increase of ≥50 points in the H-score between transurethral resection (TUR) specimens and matched RC specimens. Conversely, upregulation (−) was defined as an increase of <50 points in the H-score, no change, or a decrease in the H-score between the paired specimens. CSS and OS curves were generated using the Kaplan-Meier method and compared using the log-rank test.

In RC specimens, HMGB1 expression scores of 0-1 and 2-3 were defined as low and high, respectively, whereas calreticulin expression scores of 0-2 and 3 were defined as low and high, respectively. The HMGB1 high-expression group exhibited significantly longer CSS and OS (P < 0.01 for both; Figure 4A, 4B), while the calreticulin high-expression group demonstrated significantly longer OS (P = 0.02; Figure 4C, 4D).

Among patients who exhibited upregulation of both HMGB1 and calreticulin between TUR and matched RC specimens, only upregulation of HMGB1 was significantly associated with prolonged CSS and OS (P < 0.01 and P = 0.02, respectively; Figure 4E, 4H).

Univariable analysis revealed that pT stage ≥ pT3, histological subtype, and upregulation of HMGB1 were significantly associated with cancer-specific death. In multivariable analysis, histological subtype (hazard ratio [HR], 5.84; 95% confidence interval [CI], 1.01-33.9; P = 0.05) and upregulation of HMGB1 (HR, 0.10; 95% CI, 0.01-0.80; P = 0.03) remained significantly associated with cancer-specific death (Table 2).

**Table 2:** Univariable and multivariable analyses of prognostic factors for cancer-specific and all-cause death in all 45 patients. Calreticulin and HMGB1 upregulation in transurethral resection and radical cystectomy specimens. NAC, neoadjuvant chemotherapy; HMGB1, high-mobility group box 1.

		Cancer-specific death						All-cause death						
		Univariable			Multivariable			Univariable			Multivariable			
Factor		HR	95 % CI	P	HR	95 % CI	P	HR	95 % CI	P	HR	95 % CI	P	
Age	Less than 70 yo	ref	-	-	-	-	-	ref	-	-	-	-	-	
	70 yo or more	0.65	0.15-2.92	0.52	-	-	-	0.77	0.18-3.16	0.76	-	-	-	
Gender	Male	ref	-	-	-	-	-	ref	-	-	-	-	-	
	Female	3.16	0.55-19.52	0.14	2.49	0.20-30.5	0.48	1.39	0.25-8.22	0.72	-	-	-	
pT stage	Less than pT3	ref	-	-	-	-	-	ref	-	-	-	-	-	
	pT3 or more	9.26	1.65-99.15	<0.01	6.77	0.96-47.6	0.05	4.01	1.01-17.8	0.04	3.33	0.84-13.3	0.09	
pN stage	Negative	ref	--	-	-	-	-	ref	-	-	-	-	-	
	Positive	2.8	0.63-13.06	0.17	2.24	0.26-19.4	0.46	1.07	0.25-4.54	1	-	-	-	
Histological subtype	No	ref	-	-	-	-	-	ref	-	-	-	-	-	
	Yes	4.58	1.04-24.76	0.03	5.84	1.01-33.9	0.05	4.71	1.19-21.0	0.02	4.48	1.13-17.6	0.03	
Neoadjuvant chemotherapy	No	ref	-	-	-	-	-	ref	-	-	-	-	-	
	Yes	0.59	0.14-2.43	0.53	-	-	-	0.65	0.17-2.45	0.55	-	-	-	
Adjuvant therapy	No	ref	-	-	-	-	-	ref	-	-	-	-	-	
	Yes	1.37	0.10-13.6	1.00	-	-	-	1.35	0.14-17.78	1.00	-	-	-	
Calreticulin upregulation	No	ref	-	-	-	-	-	ref	-	-	-	-	-	
	Yes	0.73	0.14-3.36	0.74	-	-	-	1.24	0.29-5.49	0.76	-	-	-	
HMGB1 upregulation	No	ref	-	-	-	-	-	ref	-	-	-	-	-	
	Yes	0.14	0.01-0.79	0.01	0.10	0.01-0.80	0.03	0.26	0.06-1.04	0.04	0.30	0.07-1.22	0.09	

For all-cause mortality, univariable analysis revealed that pT stage ≥ pT3, histological subtype, and upregulation of HMGB1 were significantly associated with death from any cause. In multivariable analysis, only histological subtype (HR 4.48, 95% CI 1.13-17.6, P = 0.03) remained significantly associated with all-cause mortality, whereas upregulation of HMGB1 and pT stage ≥ pT3 (HR, 0.30; 95% CI, 0.07-1.22; P = 0.09 and HR, 3.33; 95% CI, 0.84-13.3; P = 0.09, respectively) showed a trend toward association (Table 2).

Upregulation of ICD-related proteins and outcomes of immune checkpoint inhibitor therapy in recurrent bladder cancer after RC

**Figure 5:**
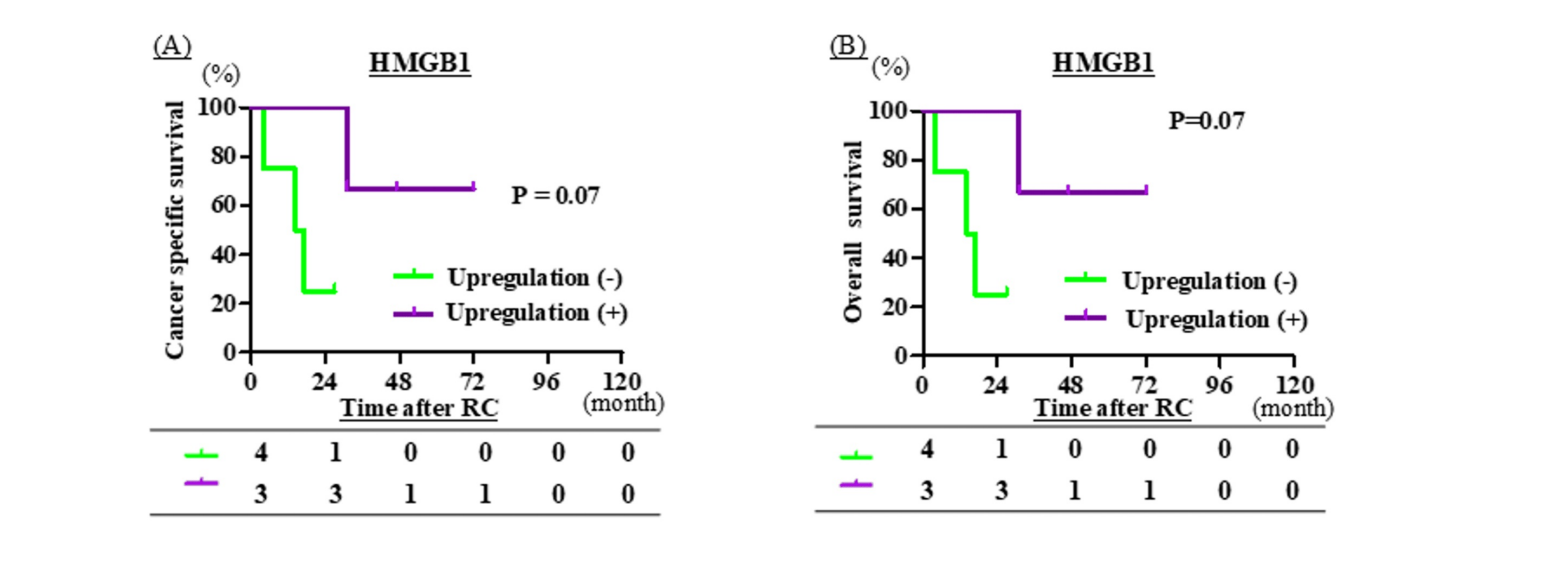
(A–B) Comparison of high-mobility group box 1 (HMGB1) upregulation between transurethral resection of bladder tumor (TURBT) and radical cystectomy (RC) specimens in seven patients who received immune checkpoint inhibitors after neoadjuvant chemotherapy (NAC) and RC. (A, B) Comparison of HMGB1 upregulation, and (C, D) comparison of calreticulin upregulation, between transurethral resection (TUR) of the TURBT and RC specimens in seven patients who received immune checkpoint inhibitor therapy after neoadjuvant chemotherapy and RC. Upregulation (+) of HMGB1 and calreticulin was defined as an increase of ≥50 points in the H-score between TUR specimens and matched RC specimens. Conversely, upregulation (−) was defined as an increase of <50 points in the H-score, no change, or a decrease in the H-score between the paired specimens. Cancer-specific survival and overall survival curves were generated using the Kaplan-Meier method and compared using the log-rank test.

A total of 26 patients experienced recurrence after RC, among whom 19 received treatment for recurrent disease. Of these, seven patients who had undergone NAC before RC received immune checkpoint inhibitor therapy. In this subgroup, patients with upregulated HMGB1 expression demonstrated a trend toward prolonged CSS and OS (P = 0.07 for both; Figure 5).

## Discussion

In this study, we investigated the induction of ICD in patients with MIBC who underwent RC with or without NAC. Our results demonstrated that NAC induced significant upregulation of HMGB1 and calreticulin expression in RC specimens, whereas no significant changes were observed in patients who did not receive NAC. Notably, upregulation of HMGB1 was associated with improved CSS and OS, suggesting its potential as a prognostic biomarker of chemotherapy-induced ICD.

The release of DAMPs, such as HMGB1 and calreticulin, represents a hallmark of ICD and plays a crucial role in activating adaptive antitumor immunity. Our findings are consistent with preclinical evidence demonstrating that chemotherapy can convert dying tumor cells into immunogenic entities, thereby promoting dendritic cell maturation and tumor-specific T-cell responses [9,11-13]. Supporting this, previous clinical studies in breast cancer and esophageal squamous cell carcinoma have reported that NAC alone can significantly upregulate HMGB1 and calreticulin expression in tumor tissues, indicating ICD induction even in the absence of radiotherapy [17]. The significant post-NAC increase in HMGB1 and calreticulin expression observed in RC specimens in our study further suggests that NAC effectively induced ICD in bladder cancer, which may have contributed to the improved survival outcomes in these patients.

Interestingly, among the NAC regimens evaluated in our cohort, upregulation of HMGB1 and calreticulin was predominantly observed in patients treated with gemcitabine-based regimens, although no statistically significant differences were detected among the regimens. This lack of significance may be attributable, at least in part, to the relatively small sample size within each treatment group, which could have limited the statistical power to detect subtle differences. Nevertheless, this observation aligns with preclinical and clinical findings indicating that chemotherapeutic agents vary in their capacity to induce ICD and modulate antitumor immunity. For instance, preclinical studies have demonstrated that certain agents, including anthracyclines and oxaliplatin, exhibit strong immunogenic potential, whereas others exert weaker effects [11,13]. Clinical evidence indicates that chemotherapy can promote dendritic cell maturation and tumor-specific T-cell responses, supporting the concept that the type of drug and composition of the regimen may influence the extent of ICD induction. Preclinical studies have demonstrated that certain agents, including anthracyclines and oxaliplatin, exhibit strong immunogenic potential, whereas others exert weaker effects [11,13]. Similarly, clinical studies have shown that chemotherapy can enhance dendritic cell maturation and tumor-specific T-cell activation, reinforcing the notion that the drug class and regimen design may modulate the degree of ICD induction [17]. Collectively, these findings suggest that the selection and combination of chemotherapeutic agents may shape the immune landscape within the tumor microenvironment and ultimately influence the clinical efficacy of NAC. In addition, in our cohort, patients with recurrent BC who received NAC followed by RC and subsequent immune checkpoint inhibitor therapy tended to exhibit improved survival when HMGB1 was upregulated. This observation suggests that chemotherapy-induced HMGB1 upregulation may enhance antitumor immune activation and sensitize tumors to subsequent immunotherapy. Although this trend did not reach statistical significance, likely owing to the small sample size, the findings imply a potential mechanistic link between ICD induction and favorable responses to immune checkpoint inhibition in recurrent BC.

In the present study, among the evaluated ICD markers, only upregulation of HMGB1 was significantly associated with improved CSS and OS in patients who received NAC. This finding underscores the pivotal role of HMGB1 in mediating chemotherapy-induced antitumor immune responses. Previous studies have identified HMGB1 as a prognostic factor in several malignancies. For instance, Wang et al. demonstrated in a meta-analysis that HMGB1 overexpression is generally associated with poor OS, whereas Guo-Liang Yang et al. reported that increased HMGB1 expression correlates with poor prognosis in human BC [18,19]. Interestingly, in the context of NAC, HMGB1 upregulation was associated with improved CSS in our cohort. This apparent discrepancy may be attributed to the dual role of HMGB1: chronic overexpression in untreated tumors may promote tumor progression and immune evasion, whereas acute HMGB1 release induced by chemotherapy functions as an immunogenic signal that enhances dendritic cell activation and tumor-specific T-cell responses. Although both calreticulin and HMGB1 are involved in ICD, they act at different stages of the immune response. Calreticulin primarily functions as an early phagocytic “eat-me” signal, whereas HMGB1 reflects downstream immune activation and antigen presentation, processes that are more directly linked to sustained antitumor immunity and long-term survival. Therefore, calreticulin expression alone may not be sufficient to indicate durable immune-mediated tumor control, which may explain why HMGB1, but not calreticulin, correlated with prognosis in our study. Therefore, HMGB1 upregulation in our study likely reflects effective induction of ICD, which may have contributed to the observed improvement in CSS. These results suggest that assessment of HMGB1 expression in BC specimens could complement conventional pathological evaluation and serve as a surrogate marker for chemotherapy-induced ICD, providing potential guidance for optimizing NAC regimens.

Several limitations of this study should be acknowledged. First, the study was retrospective and involved a relatively small sample size, which may limit the generalizability of the findings. Second, although the semi-quantitative histochemical (H)-score method is widely used, it remains subject to interobserver variability. Third, functional immune responses were not directly evaluated; therefore, the relationship between DAMP expression and the actual activation of antitumor immunity remains inferential. In addition, cases in which tumors had completely regressed after RC were excluded, which may have influenced the interpretation of the results. Moreover, no patients in this cohort received adjuvant immune checkpoint inhibitor therapy, limiting the ability to assess the relationship between ICD-related markers and clinical outcomes in the context of contemporary adjuvant immuno-oncology strategies. Finally, the follow-up period was relatively short, and longer observation is needed to comprehensively evaluate the long-term impact of ICD on survival outcomes.

## Conclusions

In conclusion, our findings indicate that NAC induces ICD in BC, as evidenced by increased HMGB1 and calreticulin expression in RC specimens. Upregulation of HMGB1 was associated with favorable survival outcomes, suggesting its potential utility as a prognostic biomarker and surrogate indicator of chemotherapy-induced immunogenicity. Future prospective studies with larger patient cohorts and detailed functional immune analyses are warranted to validate these findings and further elucidate the therapeutic implications of ICD induction in BC management.
